# Expression of key regulatory genes in necroptosis and its effect on the prognosis in non-small cell lung cancer

**DOI:** 10.7150/jca.46172

**Published:** 2020-07-11

**Authors:** Ji Eun Park, Jang Hyuck Lee, Shin Yup Lee, Mi Jeong Hong, Jin Eun Choi, Sunji Park, Ji Yun Jeong, Eung Bae Lee, Sun Ha Choi, Yong Hoon Lee, Hye won Seo, Seung Soo Yoo, Jaehee Lee, Seung Ick Cha, Chang Ho Kim, Jae Yong Park

**Affiliations:** 1 Department of Internal Medicine, School of Medicine, Kyungpook National University, Daegu, Republic of Korea.; 2 Department of Biochemistry, School of Medicine, Kyungpook National University, Daegu, Republic of Korea.; 3 BK21 Plus KNU Biomedical Convergence Program, Department of Biomedical Science, Kyungpook National University, Daegu, Korea.; 4 Cell and Matrix Research Institute, School of Medicine, Kyungpook National University, Daegu, Korea.; 5 Department of Pathology, School of Medicine, Kyungpook National University, Daegu, Republic of Korea.; 6 Department of Thoracic Surgery, School of Medicine, Kyungpook National University, Daegu, Republic of Korea.

**Keywords:** necroptosis, RIPK1, RIPK3, MLKL, NSCLC, prognosis

## Abstract

**Background:** Accumulating evidence suggests that necroptosis, or programmed necrotic cell death, may play a significant role in cancer. We evaluated the expression of key molecules in necroptosis and their association with clinical features and prognosis in NSCLC.

**Methods:** A total of 253 NSCLC patients (96 squamous cell carcinoma [SCC] cases and 157 adenocarcinoma [AC] cases) who underwent curative resection were included. Tumor tissues and corresponding normal tissues were investigated for relative mRNA expression levels of *RIPK1*, *RIPK3*, and *MLKL*. Difference in disease free survival (DFS) was analyzed according to the expression levels of these molecules in tumor tissues.

**Results:** NSCLC tissues had significantly lower expression of *RIPK1*, *RIPK3*, and *MLKL* than normal tissues (*P* = 1 x 10^-4^, *P* = 8 x 10^-6^, and *P* = 4 x 10^-8^, respectively). In subgroup analysis, SCCs had significantly lower *RIPK1*, *RIPK3*, and *MLKL* expression (*P* = 5 x 10^-4^, *P* = 3 x 10^-15^, *P* = 1 x 10^-5^, respectively), and ACs had significantly lower *RIPK1* and *MLKL* expression (*P* = 0.01 and *P* = 6 x 10^-4^, respectively) than normal tissues. Low expression of *RIPK1*, *RIPK3*, and *MLKL* in tumors was associated with a worse DFS (HR = 1.71, *P* = 0.01; HR = 1.53, *P* = 0.04; and HR = 1.53, *P* = 0.04, respectively) in a multivariate analysis. In SCC, none of the *RIPK1*, *RIPK3*, and *MLKL* expression was significantly associated with DFS. However, in AC, low expression of *RIPK1*, *RIPK3*, and *MLKL* was significantly associated with worse DFS (HR = 1.67, *P* = 0.03; HR = 1.70, *P* = 0.03; and HR = 1.81, *P* = 0.02, respectively).

**Conclusions:** Key regulatory genes in necroptosis, *RIPK1*, *RIPK3*, and *MLKL*, were downregulated in NSCLC, and their lower expression in NSCLC may be used to predict early recurrence after curative resection, especially in AC.

## Introduction

Necroptosis is a programmed necrotic cell death that is mechanistically similar to apoptosis and morphologically similar to necrosis 1. In forms of necrotic cell death, necroptosis induces the organelle swelling, rupture of cell membrane and leak of their contents into the intercellular space. But unlike necrosis, the cell lysis in necroptosis is tightly regulated 2. The key regulatory molecules of necroptosis include receptor-interacting protein kinase 1 (RIPK1), RIPK3, and mixed-lineage kinase domain like pseudokinase (MLKL), which in together form a necrosome complex 3. After receiving cell death stimuli, ubiquitination of RIPK1 is a crucial regulatory step in determining cell fate between cell survival and apoptotic or necroptotic cell death. Deubiquitination of RIPK1 induces the phosphorylation of RIPK1 and RIPK3 complex, mediating MLKL phosphorylation which is the key event in the execution of necroptosis 2. Membrane localization of MLKL induces cell membrane rupture leading to tissue inflammation and organ injury 4. There is evidence that this regulated inflammatory cell death pathway plays an important pathophysiological role in inflammatory diseases, cardiovascular diseases, neurodegenerative diseases, and malignancies 3,5,6. As a tumor suppressor effect necroptosis serves as a “fail-safe” mechanism that protects against tumor development when apoptosis is compromised 1. In contrast, as necroptosis is naturally an inflammatory mode of cell death, this pathway may induce a tumor-promoting inflammatory microenvironment which ultimately accelerates malignant transformation and cancer progression 7. Until now, only small numbers of studies have investigated the role of necroptosis in lung cancer.

Despite the current advances in anti-cancer therapies, lung cancer is still the leading cause of cancer-related deaths 8. Approximately 85% of lung cancers are non-small cell lung cancers (NSCLCs), and surgery remains the only potentially curative treatment for early NSCLC patients 9. However, 30-55% of patients with resected NSCLC experience tumor recurrence and eventually die of their disease 10. Currently, tumor stage is the strongest predictor of the prognosis of lung cancer but the prognosis of patients even in the same stages often varies widely 11. For this reason, novel prognostic biomarkers for predicting the early recurrence after the curative resection would help clinicians to have better plans for adjuvant treatment and surveillance.

The aim of this study was to evaluate the expression of the key regulatory genes - *RIPK1*, *RIPK3*, and *MLKL* - in surgically resected NSCLC and to investigate their association with clinical features and surgical outcome.

## Materials and Methods

### Patients

A total of 253 patients with available fresh frozen tumors and paired nonmalignant lung tissues who were diagnosed with pathologic stages I, II, or IIIA (micro-invasive N2) NSCLC and underwent curative surgical resection at Kyungpook National University Chilgok Hospital (KNUCH) were enrolled in this study. All patients included in this study were ethnic Koreans. Tumor and paired normal lung tissue samples from the patients were provided by the National Biobank of Korea — KNUCH, which is supported by the Ministry of Health, Welfare and Family Affairs. All materials derived from the National Biobank of Korea —KNUCH were obtained under institutional review board-approved protocols. None of the patients received chemotherapy or radiotherapy prior to surgery. Written informed consent was obtained from all patients prior to surgery. This study was approved by the institutional review boards of KNUCH, and was performed in accordance with the research protocol which was approved by the institutional review boards of KNUCH.

### Quantitative reverse transcription-polymerase chain reaction (RT-PCR)

*RIPK1*, *RIPK3* and *MLKL* mRNA expression level was measured by Quantitative RT-PCR using 253 pairs of tumor and corresponding normal lung tissues. Total RNA were isolated from fresh frozen tumors and paired nonmalignant lung tissues of the patients using Trizol (Invitrogen, Carlsbad, USA) and reverse transcribed using the QuantiTect reverse transcription kit (QIAGEN, Hilden, Germany) according to the manufacturer's instructions. Real time-PCR was performed for each gene and beta-actin with QuantiFast SYBR Green PCR Master Mix (QIAGEN) in a LightCycler 480 (Roche Applied Science, Mannheim, Germany) using the following primers: *RIPK1* forward, 5'- GCAGTTGTGAAGAGAATGCAG -3'; *RIPK1* reverse, 5'-GAAGGAGCAAACCAGGACTC -3';* RIPK3* forward, 5'- AACTGGAACACCAAGTCCTG -3'; *RIPK3* reverse, 5'- CACCCCAGAGCAGTTGTATATG -3'; *MLKL* forward, 5'- TTCGAATCTCCCAACATCCTG -3'; *MLKL* reverse, 5'- TTTTCCCTATCCAACAGCTCC -3'; beta-actin forward, 5′- TTGTTACAGGAAGTCCCTTGCC -3′; beta-actin reverse, 5′- ATGCTATCACCTCCCCTGTGT -3′. The relative mRNA expression were normalized with beta-actin expression and then calculated by the2^-∆∆CT^ method.

### Statistical analysis

We used the Student *t*-test for comparisons of continuous variables, and the chi-square or Fisher's exact test for comparisons of categorical variables. The relationship between three necroptotic molecules was investigated using Pearson correlation analysis. For survival analyses, we defined disease-free survival (DFS) as the interval from surgery to the first evidence of disease recurrence or last evaluation. The Kaplan-Meier analysis and log-rank tests were used to evaluate the differences in DFS. The Cox proportional hazard model was used for multivariate survival analyses. The hazard ratio (HR) and 95% confidence interval (CI) were estimated. All tests for significance were two-sided, and all variables with a *P*-value of less than 0.05 were considered to be significant. All statistical analyses were undertaken on SPSS version 25.0 (IBM Corporation, Armonk, NY, USA).

## Results

A total of 253 cases of NSCLC - 96 squamous cell carcinomas (SCCs) and 157 adenocarcinomas (ACs) - were included in this study. We measured relative mRNA expression levels of *RIPK1*, *RIPK3*, and *MLKL* in tumor and corresponding normal lung tissues. Table 1 shows the association between the expression level of key necroptotic genes and clinical characteristics. For this analysis, the patients were divided into high and low expression groups by median expression level. Low expression of *RIPK3* was significantly associated with the male gender (*P*=6×10^-5^), SCC (*P*=2×10^-4^), and smoker (*P*=0.003). Low expression of *MLKL* was significantly associate with AC (*P*=0.04). However, *RIPK1* expression was not associated with sex, histology, smoking status, and adjuvant chemotherapy. None of the three genes were associated with the pathologic stage. In AC, smoking status and *EGFR* mutation status was not associated with the expression of those genes.

**Figure 1:**
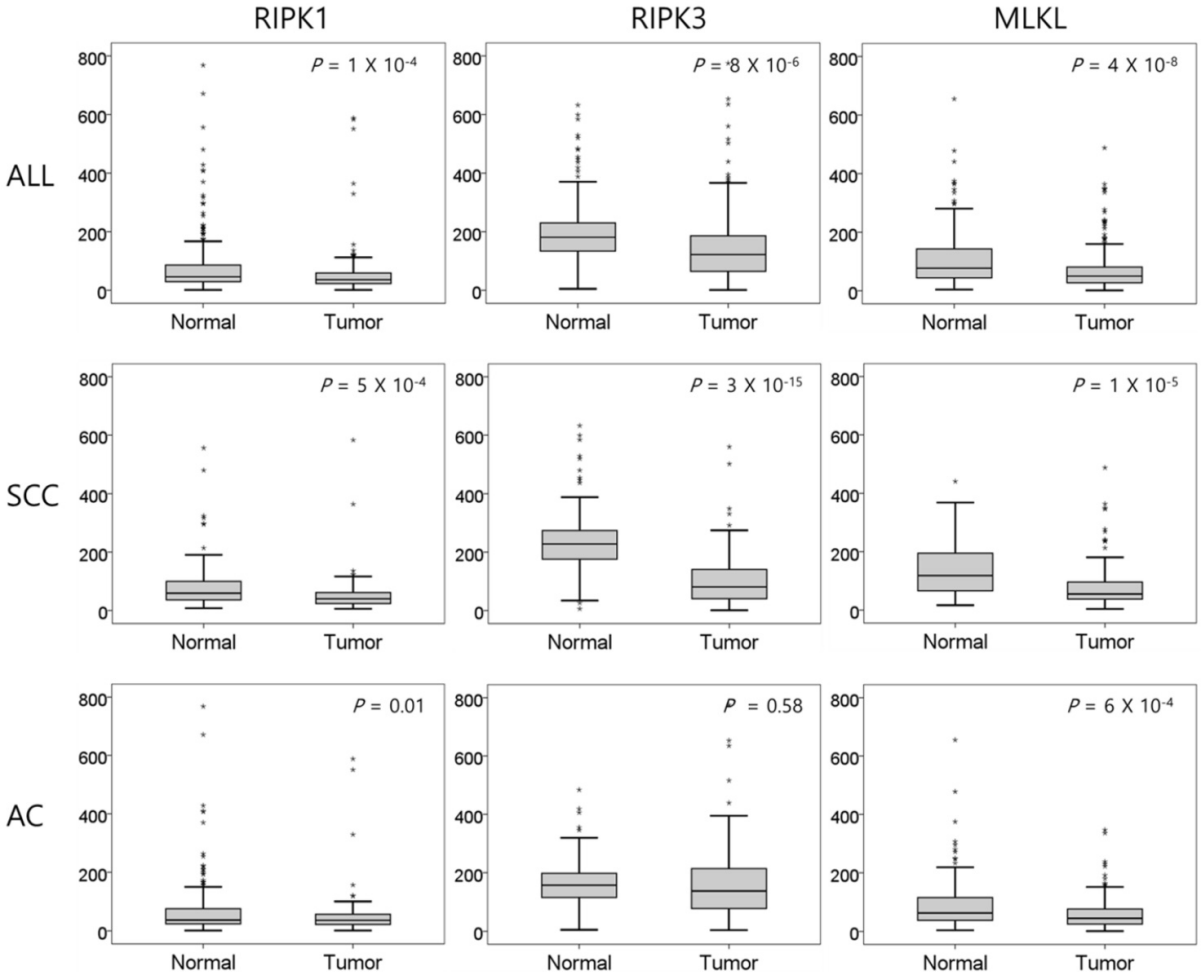
Comparison of relative mRNA expression levels of *RIPK1*, *RIPK3*, and *MLKL* in normal lung tissues and tumor tissues. SCC, squamous cell carcinoma; AC, adenocarcinoma. *P* values by the paired t-test.

**Figure 2:**
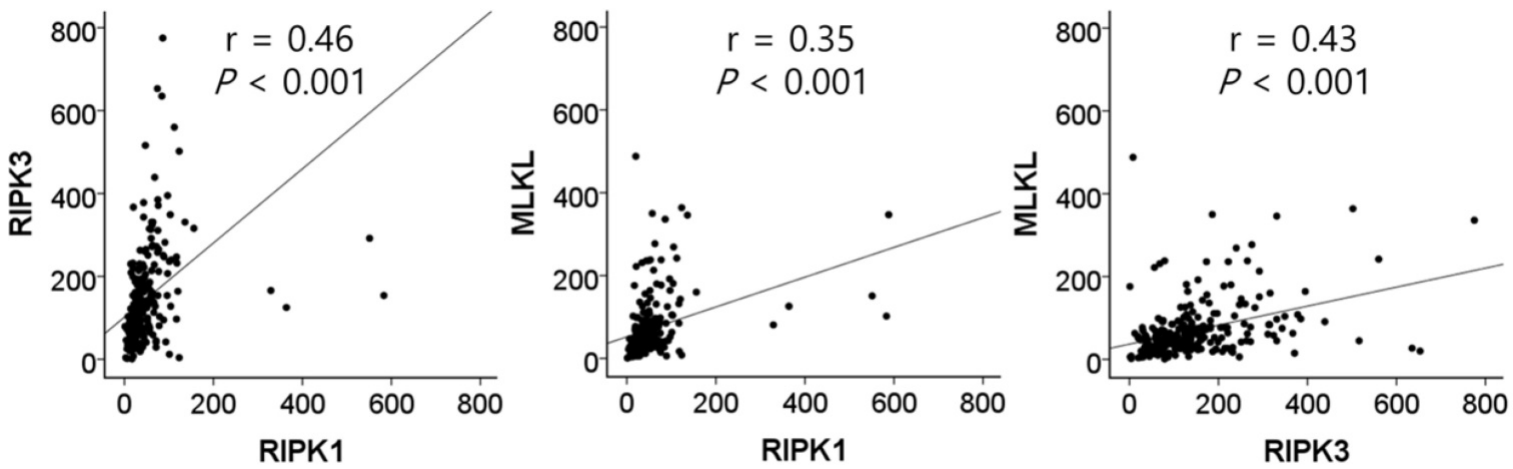
Correlation between the mRNA expression of *RIPK1*, *RIPK3*, and *MLKL* in tumor tissues by Pearson correlation analysis.

The mRNA expression of *RIPK1*, *RIPK3*, and *MLKL* was significantly lower in NSCLC tissues than in normal lung tissues (*P* = 1 × 10^-4^, *P* = 8 × 10^-6^, and *P* = 4 × 10^-8^, respectively, Figure 1). In SCCs, tumor tissues had significantly lower mRNA expression level of *RIPK1* (*P* = 5 × 10^-4^), *RIPK3* (*P* = 3 × 10^-15^), and *MLKL* (*P* = 1 × 10^-5^) compared to normal lung tissues. In addition, ACs had significantly lower mRNA expression level of *RIPK1* (*P* = 0.01) and *MLKL* (*P* = 6 × 10^-4^) compared to normal tissues. The mRNA expression of *RIPK1*, *RIPK3*, and *MLKL* in tumor tissues was significantly correlated with each other (r = 0.46, *P* <0.001, between *RIPK1* and *RIPK3*; r = 0.35, *P* <0.001, between *RIPK1* and *MLKL*; r = 0.43, *P* <0.001, between *RIPK3* and *MLKL*; Figure 2).

**Figure 3:**
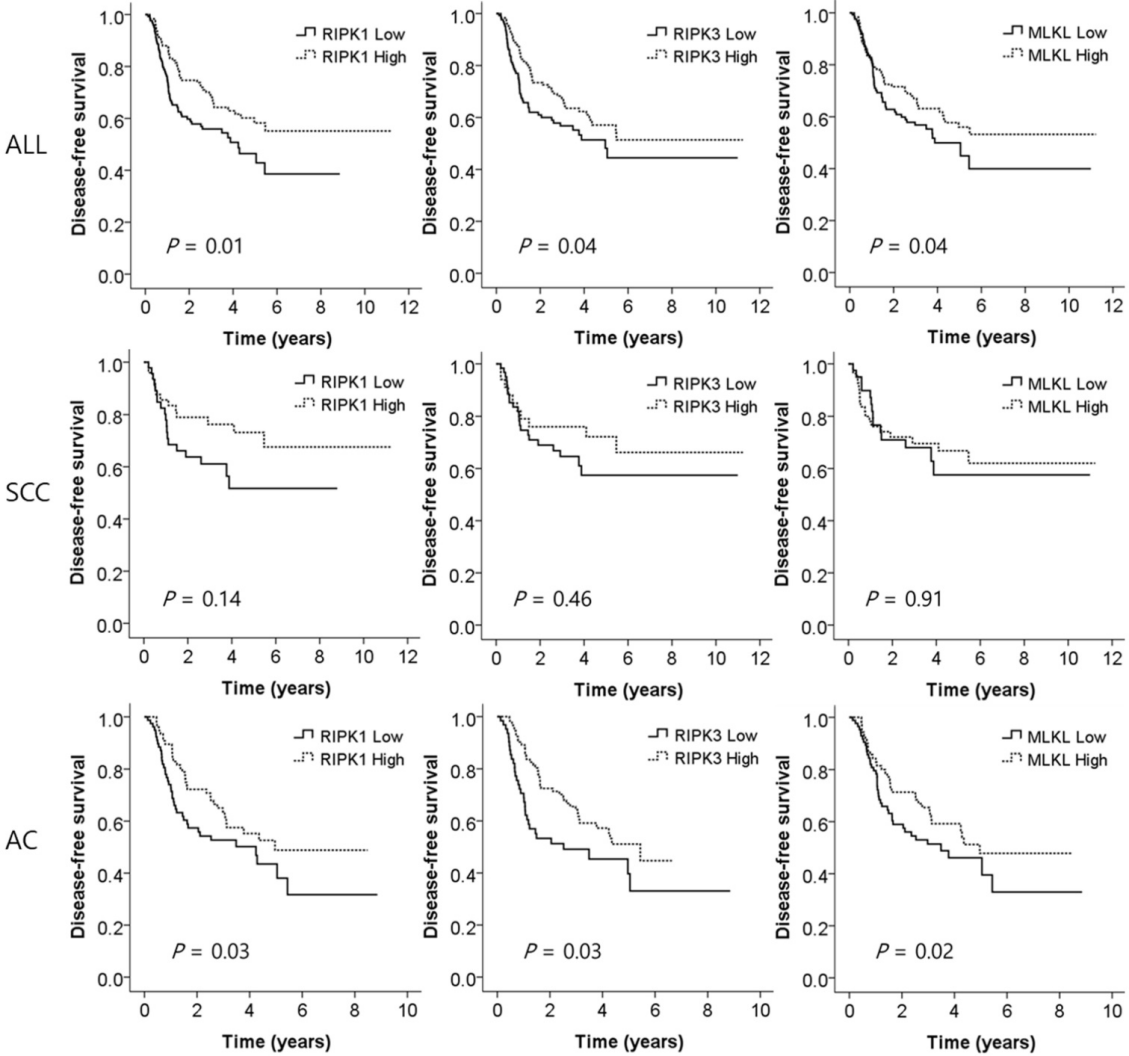
Disease-free survival curves according to the expression levels of *RIPK1*, *RIPK3*, and *MLKL*. SCC, squamous cell carcinoma; AC, adenocarcinoma. *P* values by multivariate Cox proportional hazard model.

To investigate whether the expression of the key genes of necrotopsis have prognostic role, we measured and compared DFS between low and high expression groups. Multivariate analysis demonstrated that low expression of all three key molecules was associated with a worse DFS after adjusting for age, gender, histologic type, smoking status, stage, and adjuvant chemotherapy (adjusted HR [aHR] = 1.71, 95% CI = 1.16-2.53, *P* = 0.01 for *RIPK1*; aHR = 1.53, 95% CI = 1.02-2.31, *P* = 0.04 for *RIPK3*; aHR = 1.53, 95% CI = 1.03-2.27, *P* = 0.04 for *MLKL*, Table 2 and Figure 3). Subgroup analysis showed that in SCCs, there was no significant difference in DFS between low and high expression of the genes. However, in ACs, low expression of *RIPK1*, *RIPK3*, and *MLKL* was significantly associated with early recurrence after surgical resection (aHR = 1.67, 95% CI = 1.04-2.66, *P* = 0.03; aHR = 1.70, 95% CI = 1.04-2.78, *P* = 0.03; aHR = 1.81, 95% CI = 1.11-2.96, *P* = 0.02, respectively, Table 2 and Figure 3).

Because necroptosis is one of the molecular mechanisms of necrosis, we analyzed the correlation between the expression of *RIPK1*, *RIPK3*, and *MLK1*, and the presence of necrotic area. For this analysis, we reviewed pathological reports of 137 patients which were available. Among them, 59 patients (43.1%) had tumor necrosis. The mRNA expression levels of *RIPK1* and *RIPK3* were significantly lower in patients with tumor necrosis (*P* = 0.04 and* P* = 2 × 10^-5^, respectively) than in those without tumor necrosis (Supplementary Figure 1). *MLKL* expression was also lower in patients with necrosis, although not significant (*P* = 0.24).

## Discussions

In this study, we investigated the expression of key regulators of necroptosis and their association with clinical features and surgical outcome in NSCLC. We showed that the expression of *RIPK1*, *RIPK3* and *MLKL* was significantly lower in tumors compared to normal lung tissues, and that the patients with decreased expression of the genes in tumors had significantly worse DFS. Subgroup analysis showed that the effect of low expression of the genes was more prominent in AC. The results suggest that the necroptosis pathway play important roles in the pathogenesis of NSCLC. The measurement of *RIPK1*, *RIPK3* and *MLKL* expression may be a useful method to help establish more effective management and follow-up strategies for the patients with NSCLC after surgical resection.

There have been controversies on whether necroptosis inhibits or induces tumor development. In addition to the tumor suppressor function of necroptosis, emerging data indicate that necroptosis plays a crucial role in tumorigenesis, tumor progression, and regulation of tumor immunity 1. Until now, the net effect of necroptosis in cancer remains undefined and pro- or anti-tumoral effects of necroptosis are likely to rely on the cell type and stage of the cancer 4. Feng et al. demonstrated that colorectal cancer tissues had a significantly decreased *RIPK3* expression level compared to normal tissues and that low *RIPK3* expression in tumors was a predictor of worse overall survival (OS) and DFS 12. It was reported that *RIPK1* expression was significantly downregulated in head and neck squamous cell carcinoma, and low expression in tumors was associated with progression of the disease 13. Another study in gastric cancer revealed that *MLKL* expression was significantly low in tumor tissues and was associated with decreased OS 14. These results were in agreement with our results, suggesting tumor suppressor function of necroptotic molecules in these cancers. However, different role of necroptosis has been reported in various cancers. The study by Seifert et al. reported that in pancreatic ductal adenocarcinoma, the necroptotic molecules were associated with tumor promoting function associated with macrophage-induced adaptive immune suppression 15. Recently, necroptotic cell death pathway was shown to be involved in tumor necrosis and high level of potassium released from necrotic tumor cells inhibits both CD4 and CD8 T cell activity leading to tumor development and metastasis 16,17. Another study regarding mouse MMVT-PyMT breast tumor showed that *RIPK3* expression was decreased in the early stages, but a significant increase of *RIPK3* and *MLKL* expression was detected in late stage tumors that bear tumor necrosis, suggesting the necroptosis promotes tumor development 17,18. Collectively, the results regarding the role of necroptosis in cancers are conflicting due to the pleiotropic role of necroptotic mediators, and the distinct tumor microenvironment of each type of the cancer 1.

Interestingly, tumors with necrosis had significantly lower mRNA expression levels of necroptotic molecules compared to those without necrosis. Tumor necrosis is a result of inadequate vascularization and subsequent metabolic stress such as hypoxia and nutrient deprivation 17, and it has been reported as an indicator of poor prognosis in NSCLC 19. Our study also showed a tendency toward worse DFS in patients with necrosis (Supplementary Figure 2). Because different molecular mechanisms, such as necroptotis, pyroptosis, and ferroptosis, alone or in combination, may contribute to tumor necrosis 20, the expression levels of necroptotic molecules may not correlate with the tumor necrosis. In this study, moreover, patients with low tumor expression of *RIPK1*, *RIPK3*, and *MLKL* proved to have worse DFS. Taken together, we observed an inverse relationship between the expression of necroptotic molecules and necrosis in NSCLC. These results may be a supporting evidence for the role of necroptosis as a tumor suppressor rather than as a molecular mechanism associated with tumor necrosis in early stage NSCLC.

Another interesting finding in this study is that high expression of all the three genes in normal lung tissues was significantly associated with male sex, squamous cell carcinoma, and smoking (Supplementary Table 1). This finding suggests that cigarette smoking caused the increased expression of necroptosis molecules, because smoking rates exceed 90% in male (92.5%) and squamous cell carcinoma (97.9%) in marked contrast to those in female (10%) and adenocarcinoma (47.1%) in this study, which is in agreement with smoking rates in Korean lung cancer patients 21. Cigarette smoking has been correlated with increased necroptosis 22, 23. Recent data indicate that cigarette smoking-induced necroptosis of airway epithelial cells potentially plays a role in COPD pathogenesis 22, 23. However, we observed reduced expression of necroptosis molecules in tumors compared to normal lung tissues, and lower tumor expression of *RIPK3* in smokers compared to nonsmokers in this study. Therefore, it may be speculated that cigarette smoking-induced necroptosis may play different roles in cancers and inflammatory diseases, or in different stages of carcinogenesis.

There are a few studies on the clinical implication of necroptosis in NSCLC. Recently, Wang et al. investigated the role of the necroptosis pathway in the responsiveness of NSCLC to chemotherapy 24. In the study, *RIPK3* expression was suppressed in NSCLC tumors compared with normal lung tissues, and reduced *RIPK3* expression in tumors was significantly associated with poor chemotherapy response. They also showed that reduced *RIPK3* expression in NSCLC cells was associated with promoter methylation, and that restoration of *RIPK3* expression sensitized cisplatin cytotoxicity through potentiation of necrotopsis. Chung et al. investigated *RIPK3* expression in 50 lung AC patients (Stage IB-IIIA) who had undergone surgical resection followed by platinum based adjuvant chemotherapy. They showed that high tumor expression of *RIPK3* was associated with better DFS after adjuvant chemotherapy, thus a potentially useful predictive marker for the efficacy of adjuvant chemotherapy 25. In this study we investigated three representative genes in the regulation of necroptosis, and first showed that the decreased expression of those genes may be used to predict recurrence after surgical resection in patients with NSCLC. The subgroup analysis suggested that the effect of low expression of the genes was more prominent in AC. However, although the statistical significance remained only in AC, reduced sample sizes by dividing into two groups (SCC vs. AC) may have led to type II errors. Further studies are warranted to validate our results in a larger number of patients including diverse ethnic groups.

Necroptosis is one of the mechanisms of immunogenic cell death (ICD) 26. ICD is a unique class of regulated cell death capable of eliciting complete antigen-specific adaptive immune responses, through the emission of a spatiotemporally defined set of danger signals which leads to activation and maturation of antigen presenting cells 27. Yang et al. showed that RIPK3 or MLKL deficiency abrogated the ability of dying cancer cells to secrete the required immunogenic signals that lead to an anticancer immune response in mice 28. Checkpoint blockade efficacy may be enhanced by induction of more immunogenic conditions in the tumor tissue through ICD, as corroborated by the success of combination therapy of immune checkpoint inhibitors and cytotoxic chemotherapy and/or radiotherapy 29,30. Therefore, necroptosis molecules may be a potential predictive biomarker for checkpoint blockade, which requires validation in future studies.

In conclusion, this study suggests that the necroptosis pathway play a crucial role in the pathogenesis of NSCLC. The expression of key necroptosis genes may help clinicians to identify patients at higher risk of disease recurrence in surgically resected NSCLC. Future studies are required to determine the role of necroptosis in NSCLC.

## Supplementary Material

Supplementary figures and table.

## Figures and Tables

**Table 1:** Association between clinical characteristics and the expression level of key necroptotic genes

Variables	No of patients	RIPK1			RIPK3			MLKL		
		Low	High	*P*	Low	High	*P*	Low	High	*P*
**Age**		64.9 (±9.1)	64.1(±9.3)	0.50	64.6(±9.5)	64.3(±8.9)	0.82	64.6(±9.7)	64.3(±8.7)	0.80
**Gender**										
Male	173	85(49.1)	88(50.9)	0.90	101(58.4)	72(41.6)	6×10^-5^	85(49.1)	88(50.9)	0.75
Female	80	40(50.0)	40(50.0)		25(31.2)	55(68.8)		41(51.2)	39(48.8)	
**Smoking status**										
Smoker	168	80(47.6)	88(52.4)	0.42	95(56.5)	73(43.5)	3×10^-3^	81(48.2)	87(51.8)	0.48
Non-smoker	85	45(52.9)	40(47.1)		31(36.5)	54(63.5)		45(52.9)	40(47.1)	
**Histologic type**										
SCC	96	46(47.9)	50(52.1)	0.71	62(64.6)	34(35.4)	2×10^-4^	40(41.7)	56(58.3)	0.04
AC	157	79(50.3)	78(49.7)		64(40.8)	93(59.2)		86(54.8)	71(45.2)	
Smoker	74	35(47.3)	39(52.7)	0.48	34(45.9)	40(54.1)	0.21	42(56.8)	32(43.2)	0.64
Non-smoker	83	44(53.0)	39(47.0)		30(36.1)	53(63.9)		44(53.0)	39(47.0)	
EGFR mutant	45	21(46.7)	24(53.3)	0.50	21(46.7)	24(53.3)	0.35	28(62.2)	17(37.8)	0.25
EGFR wild	89	47(52.8)	42(47.2)		34(38.2)	55(61.8)		46(51.7)	43(48.3)	
**Pathologic stage**										
Stage I	210	100(47.6)	110(52.4)	0.21	100(47.6)	110(52.4)	0.13	106(50.5)	104(49.5)	0.64
Stage II-III	43	25(58.1)	18(41.9)		26(60.5)	17(39.5)		20(46.5)	23(53.5)	
**Adjuvant CTx**										
Yes	52	26(50.0)	26(50.0)	0.92	28(53.8)	24(46.2)	0.51	24(46.2)	28(53.8)	0.56
No	201	99(49.3)	102(50.7)		98(48.8)	103(51.2)		102(50.7)	99(49.3)	

Abbreviations: RIPK1, Receptor-interacting protein kinase 1; RIPK3, Receptor-interacting protein kinase 3; MLKL, Mixed-lineage kinase domain like pseudokinase; SCC, Squamous cell carcinoma; AC, Adenocarcinoma; CTx, Chemotherapy.Data are expressed as the mean (±SD) or number (%).

**Table 2:** Multivariate analysis of disease-free survival according to the expression of three necroptotic genes (low vs high expression)

Histologic type	Genes	Log-Rank *P*	HR (95% CI)	*P*
All	*RIPK1*	0.02	1.71 (1.16-2.53)	0.01
	*RIPK3*	0.07	1.53 (1.02-2.31)	0.04
	*MLKL*	0.15	1.53 (1.03-2.27)	0.04
Squamous cell carcinoma	*RIPK1*	0.09	1.77 (0.83-3.79)	0.14
	*RIPK3*	0.32	1.36 (0.60-3.08)	0.46
	*MLKL*	0.77	1.05 (0.48-2.26)	0.91
Adenocarcinoma	*RIPK1*	0.10	1.67 (1.04-2.66)	0.03
	*RIPK3*	0.03	1.70 (1.04-2.78)	0.03
	*MLKL*	0.17	1.81 (1.11-2.96)	0.02

Abbreviations: RIPK1, Receptor-interacting protein kinase 1; RIPK3, Receptor-interacting protein kinase 3; MLKL, Mixed-lineage kinase domain like pseudokinase; HR, Hazard ratio; CI, confidence interval. HR, 95% CI and *P* values in multivariate analysis were calculated by Cox proportional hazard model, adjusted for age, gender, histologic type, smoking status, stage, and adjuvant chemotherapy.
