# Supplementary material for: Expression of key regulatory genes in necroptosis and its effect on the prognosis in non-small cell lung cancer
Source: J Cancer. 2020 Jul 11;11(18):5503–10. doi: 10.7150/jca.46172 (PMC7391199; doi:10.7150/jca.46172)
Supplement: Supplementary file 1 — Supplementary figures and table. [file jcav11p5503s1.pdf]

Supplementary Table 1. Association between clinical characteristics and the expression level of key necroptotic genes in normal lung tissues of 253 patients.

Variables	No of patients	RIPK1			RIPK3			MLKL		
		Low	High	*P*	Low	High	*P*	Low	High	*P*
Age		64.9(±8.7)	64.0(±9.7)	0.42	64.1(±9.6)	64.9(±8.7)	0.49	65.0(±9.6)	64.0(±8.7)	0.38
Gender										
Male	173	81(46.8)	92(53.2)	0.03	77(44.5)	96(55.5)	1×10^{-3}	76(43.9)	97(56.1)	2×10^{-3}
Female	80	49(61.2)	31(38.8)		53(66.2)	27(33.8)		52(65.0)	28(35.0)	
Smoking status										
Smoker	168	75(44.6)	93(55.4)	3×10^{-3}	69(41.1)	99(58.9)	4×10^{-6}	72(42.9)	96(57.1)	1×10^{-3}
Non-smoker	85	55(64.7)	30(35.3)		61(71.8)	24(28.2)		56(65.9)	29(34.1)	
Histologic type										
SCC	96	35(36.5)	61(63.5)	2×10^{-4}	28(29.2)	68(70.8)	3×10^{-8}	30(31.2)	66(68.8)	1×10^{-6}
AC	157	95(60.5)	62(39.5)		102(65.0)	55(35.0)		98(62.4)	59(37.6)	
Smoker	74	40(54.1)	34(45.9)	0.12	42(56.8)	32(43.2)	0.04	42(56.8)	32(43.2)	0.17
Non-smoker	83	55(66.3)	28(33.7)		60(72.3)	23(27.7)		56(67.5)	27(32.5)	
EGFR mutant	45	28(62.2)	17(37.8)	0.67	29(64.4)	16(35.6)	0.93	28(62.2)	17(37.8)	0.94
EGFR wild	89	52(58.4)	37(41.6)		58(65.2)	31(34.8)		56(62.9)	33(37.1)	
Pathologic stage										
Stage I	210	104(49.5)	106(50.5)	0.19	103(49.0)	107(51.0)	0.10	100(47.6)	110(52.4)	0.04
Stage II-III	43	26(60.5)	17(39.5)		27(62.8)	16(37.2)		28(65.1)	15(34.9)	
Adjuvant CTx										
Yes	52	30(57.7)	22(42.3)	0.31	25(48.1)	27(51.9)	0.59	31(59.6)	21(40.4)	0.14
No	201	100(49.8)	101(50.2)		105(52.2)	96(47.8)		97(48.3)	104(51.7)	

Abbreviations: RIPK1, Receptor-interacting protein kinase 1; RIPK3, Receptor-interacting protein kinase 3; MLKL, Mixed-lineage kinase domain like pseudokinase; SCC, Squamous cell carcinoma; AC, Adenocarcinoma; CTx, Chemotherapy.

Data are expressed as the mean (±SD) or number (%)

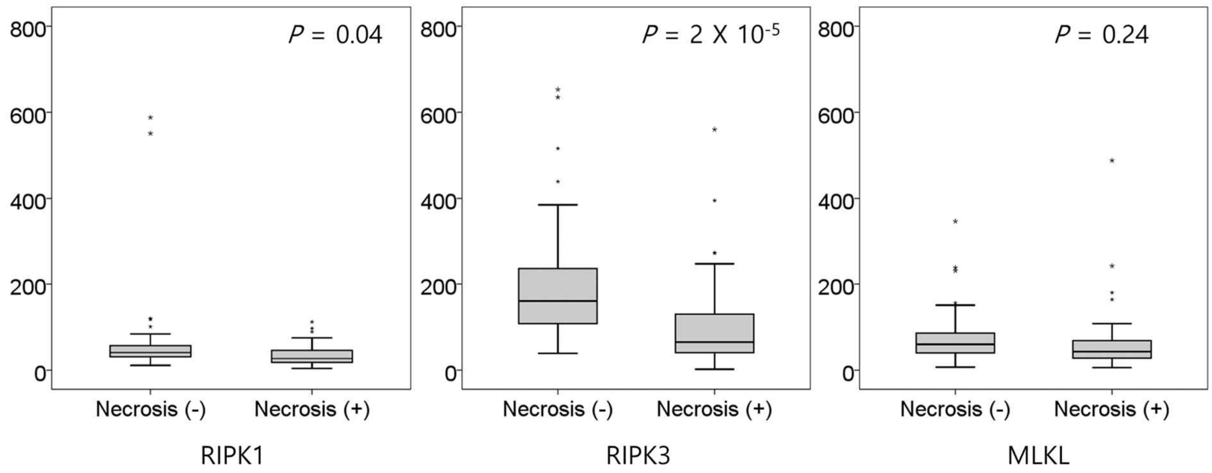

Supplementary Figure 1. Comparison of relative mRNA expression levels of *RIPK1*, *RIPK3*, and *MLKL* in tumors, according to the absence or presence of necrosis in tumors. *P* values by the Student *t*-test.

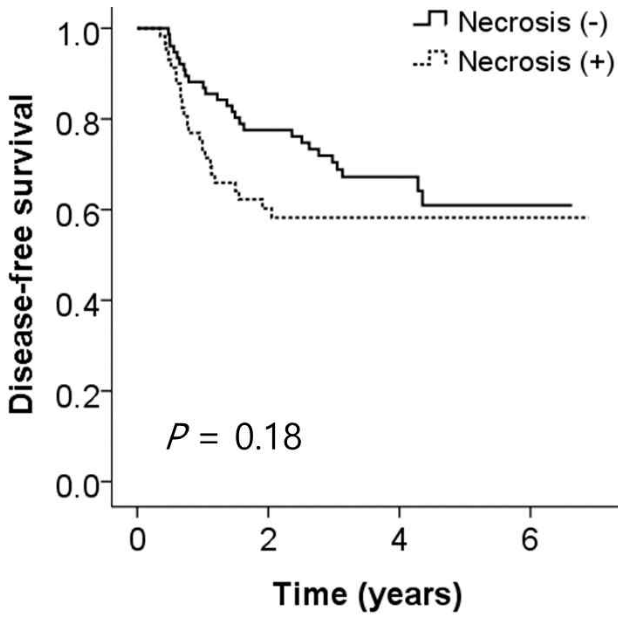

Supplementary Figure 2. Disease-free survival curve according to the absence or presence of tumor necrosis. *P* value by log rank test.
